# Marine Natural Products as Innovative Cosmetic Ingredients

**DOI:** 10.3390/md21030170

**Published:** 2023-03-08

**Authors:** Sara Fonseca, Mariana Neves Amaral, Catarina Pinto Reis, Luísa Custódio

**Affiliations:** 1 Faculty of Pharmacy, Universidade de Lisboa, Av. Prof. Gama Pinto, 1649-003 Lisboa, Portugal; 2 Research Institute for Medicines (iMed.ULisboa), Faculty of Pharmacy, Universidade de Lisboa, 1649-003 Lisboa, Portugal; 3 Instituto de Biofísica e Engenharia Biomédica (IBEB), Faculdade de Ciências, Universidade de Lisboa, Campo Grande, 1749-016 Lisboa, Portugal; 4 Centre of Marine Sciences (CCMAR), Faculty of Sciences and Technology, Campus of Gambelas, University of Algarve, Ed. 7, 8005-139 Faro, Portugal

**Keywords:** marine products, cosmetic, bioactive molecules, innovation, sustainability

## Abstract

Over the course of the last 20 years, numerous studies have identified the benefits of an array of marine natural ingredients for cosmetic purposes, as they present unique characteristics not found in terrestrial organisms. Consequently, several marine-based ingredients and bioactive compounds are under development, used or considered for skin care and cosmetics. Despite the multitude of cosmetics based on marine sources, only a small proportion of their full potential has been exploited. Many cosmetic industries have turned their attention to the sea to obtain innovative marine-derived compounds for cosmetics, but further research is needed to determine and elucidate the benefits. This review gathers information on the main biological targets for cosmetic ingredients, different classes of marine natural products of interest for cosmetic applications, and the organisms from which such products can be sourced. Although organisms from different phyla present different and varied bioactivities, the algae phylum seems to be the most promising for cosmetic applications, presenting compounds of many classes. In fact, some of these compounds present higher bioactivities than their commercialized counterparts, demonstrating the potential presented by marine-derived compounds for cosmetic applications (i.e., Mycosporine-like amino acids and terpenoids’ antioxidant activity). This review also summarizes the major challenges and opportunities faced by marine-derived cosmetic ingredients to successfully reach the market. As a future perspective, we consider that fruitful cooperation among academics and cosmetic industries could lead to a more sustainable market through responsible sourcing of ingredients, implementing ecological manufacturing processes, and experimenting with inventive recycling and reuse programs.

## 1. Introduction

Appearance and personal care play an important role in the modern lifestyle, attracting an increasing number of consumers towards products used to enhance or alter the appearance of skin, hair, and nails [1]. The cosmetics market is extremely dynamic and new products are constantly launched at an exceptionally fast rate, while new concepts are continuously emerging and new terms are being coined [2,3]. The global market for cosmetic and cosmeceutical products was forecasted to reach 463.5 billion USD by 2027 [4].

Presently, many synthetic chemicals are used in cosmetic products even though many of them do not fully meet consumers expectations [1,5,6,7]. Consequently, the demand for cosmetics containing natural ingredients is rapidly increasing, as they are promoted as green, safer, and sustainable materials [1,6,7,8].

Although classic plant-derived ingredients are still very popular and broadly used in cosmetic products, they exhibit shortcomings including slow growth and the fact that environmental and cultural practices require more investment; they also require use of arable land, which is a limited resource [2,9]. The oceans, from shallow to deep waters, encompass a wide range of habitats and environmental conditions hosting huge fauna and flora biodiversity. The unique characteristics of several marine systems have driven a variety of biological adaptations, leading to the production of a large spectrum of bioactive molecules resulting in a living library of diversity that is still unexplored and underexploited [1,2]. Moreover, marine organisms can be commercially cultivated in high quantities using modern aquaculture techniques [2].

Although there are several recent review papers containing information on the use of marine-derived bioactive products in the cosmetic industry, such studies often focus on specific groups of organisms, usually macro- and microalgae, and lack an outline of the practical challenges associated with the commercialization of such products. This review provides a combined overview of the most important biological targets for cosmetic ingredients, classes of marine natural products of interest for cosmetic applications, possible sources of such products, and pertinent challenges and opportunities related to the commercial exploitation of cosmetic ingredients from marine sources. As a future perspective, we consider that fruitful cooperation among academics and cosmetic industries could lead to a more sustainable market through responsible sourcing of ingredients, implementing ecological manufacturing processes, and experimenting with inventive recycling and reuse programs.

## 2. Materials and Methods

The search engine Google Scholar and the Web of Science database were checked to obtain the most relevant articles by using the words “cosmetic ingredients”, “marine natural products”, and/or “marine products” combined with, for example, “biological targets”, “macroalgae”, “microalgae”, “marine invertebrates”, “marine microorganisms”, “seaweeds”, “seagrasses”, “cosmetics”, and “cosmeceuticals”, and also “cosmetic legislation”, “cosmetic industry”, and “cosmetic market challenges”. Only English articles with full text were considered, preferably published in the last 10 years.

## 3. Target Biological Properties of Cosmetic Ingredients

Cosmetics are, per definition, any substance or mixture intended to be placed in contact with the external parts of the human body (epidermis, hair, nails, lips, and external genital organs) or with teeth or with the membranes of the oral cavity with the sole or main purpose to clean, perfume, change their appearance, protect, keep good condition, or alter body odors [9,10]. Cosmetic products with biologically active ingredients are formulated to improve appearance and boost positive physiological effects at the cellular level, and there is a high demand for these ingredients [2].

**Figure 1:**
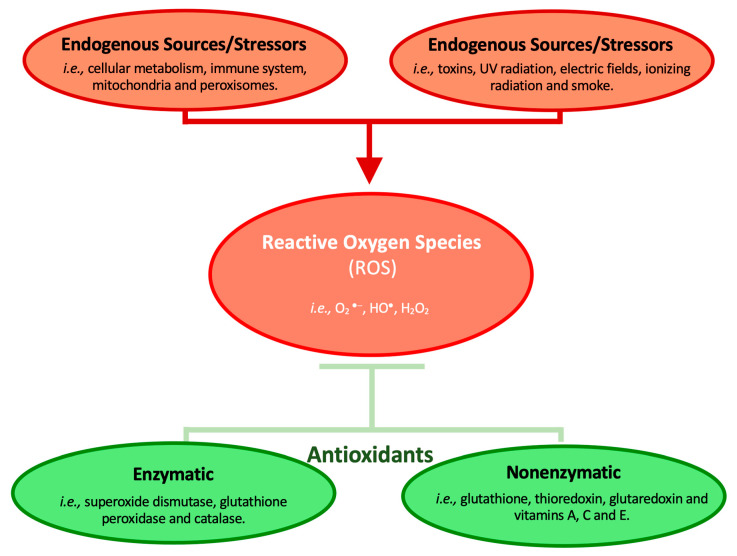
Oxidative damage and antioxidative activity, based on [38,39].

Skin is exposed to several external agents responsible for skin aging. Oxidative stress is mainly caused by reactive oxygen species (ROS) and is involved in many processes that damage the skin’s appearance by triggering cellular damage (Figure 1) [11,12]. Antioxidants consist of enzymatic (i.e., superoxide dismutase, catalase, glutathione peroxidase, glutathione reductase, and glutathione transferase) and non-enzymatic (i.e., β-carotene, R-tocopherol, ascorbic acid, and ubiquinol) molecules with several activities, including photoprotection and scavenging/immobilizing of ROS, therefore preventing damage of the lipids, proteins, and DNA [2,13]. The oxidation of membrane lipids damages the appearance of the skin, and with aging, the body’s ability to regulate ROS increases, also increasing its mitochondrial production, culminating in skin aging. Thus, to halt this process, antioxidants can be incorporated into cosmetics to lessen oxidative stress [13].

The great majority of ROS (80%) are produced in response to solar radiation, with UV rays also reducing the activity of antioxidant enzymes [3,14]. Thus, solar exposure is the biggest contributor to skin aging through hyperpigmentation and photoaging (degradation of collagen and hyaluronic acid), causing wrinkles [1,15]. Hyperpigmentation refers to the overproduction of melanin in the skin and is considered an aesthetic problem [16]. The overproduction of melanin can be transitory or permanent and promoted by many factors including, as mentioned, UV radiation [7].

Skin whitening products focus on providing equal pigmentation of the skin by decreasing melanin’s concentration, and the market value for these products is growing and expected to reach 8.9 billion USD by 2027 [7,16]. Tyrosinase is the rate-limiting enzyme involved in the synthesis of melanin and is thus a good target for skin whitening products. The cosmetic industry is expanding the use of natural depigmentation ingredients, such as liquiritin, isoliquertin, aloesin, arbutin, and vitamin C, as these have fewer side effects than synthetic components and are eco-friendly [17,18].

Over the years, awareness of the skin damage caused by UV rays and solar exposure has increased and led to the production and commercialization of cosmetics with photoprotective properties [16]. Matrix metalloproteinases (MMPs) (i.e., collagenases, gelatinases, and stromelysins) are responsible for the degradation of proteins of the extracellular matrix (i.e., collagen, elastin, and hyaluronic acid) [19,20,21]. The expression of MMPs is stimulated by UV radiation, promoting skin aging through photoaging and the formation of wrinkles. Due their involvement in photoaging processes, compounds able to inhibit MMPs are of interest for the development of cosmetics to prevent photoaging of the skin and wrinkle formation [2,22]. In fact, anti-aging products are amongst the most marketed and commercialized cosmetics in the world [13,16]. The main process involved in the aging of the skin is the degradation of the extracellular matrix in the epidermal and dermal layers of the skin. Although genetics (intrinsic aging) are detrimental, extrinsic factors (i.e., exposure to UV radiation and pollution, nicotine, and lifestyle choices) also contribute to accelerating this process [2,3,23,24]. Most anti-aging cosmetics focus on stimulating the production of proteins of the extracellular matrix, such as collagen and glycosaminoglycan, to increase the firmness and elasticity of the skin [2,25,26].

Another very important aspect to increasing the firmness and elasticity of the skin is its hydration [27]. A disruption in skin hydration may lead to accelerated desquamation, and the usual cosmetic treatments for dehydrated skin include lipid-based moisturizers to retain water by occlusion. Collagen is a common constituent of moisturizers with well-known hydrating benefits [1,12,16]. Dehydrated skin is also characterized by a loss of hyaluronic acid, decreasing the skin’s elasticity. Thus, many cosmetics aiming to treat dehydrated skin contain hyaluronic acid; however, its role in skin rehydration is still controversial, as the higher the molecular weight of hyaluronic acid, the higher its moisturizing abilities [13,28,29,30]. In fact, the cosmetic applications of hyaluronic acid appear to change in relation to its molecular weight: high-molecular-weight hyaluronic acid rehydrates the skin by contributing to osmotic balance, and consequently stabilizes the extracellular matrix; hyaluronic acid of medium molecular weight enhances wound healing and cell repair by modulating inflammation and angiogenesis; while low-molecular-weight hyaluronic acid can be included in cosmetic formulations for both wound healing enhancement and moisturizing abilities [28,30].

Acne vulgaris is another skin disorder with significant prevalence. It is complex and multifactorial and usually associated with commensal skin microbiota, increased serum production, and hyperkeratosis [31,32,33]. *Propionibacterium acnes* and *Staphylococcus epidermidis* are the main bacteria involved, leading to the production of proinflammatory cytokines and the release of ROS, whose excessive production results in a destructive phenomenon, leading to scarring [2,34]. These bacteria also release lipases to digest the surplus of skin oil and sebum, which in turn stimulates an intense local inflammation that bursts from the hair follicles [35]. Therefore, the inhibition of both bacteria has been recognized as a strategic method for the management of acne in the cosmetics industry [2,32,35,36,37]. Acne vulgaris is conventionally treated with antibiotics, such as clindamycin and erythromycin, and in some cases, it entails oral antibiotics. Yet, extensive application of antibiotics might lead to bacterial resistance. Furthermore, antibiotics may cause skin allergies and irritation [31]. Owing to the multiple factors involved in the pathogenesis of acne and the implications of the use of antibiotics, it is important to highlight, for example, the bacterial growth-inhibiting activities of some marine ingredients [33,35].

## 4. Natural Products

Natural products are products sourced or produced by plants, animals, and microorganisms. Sometimes their production might be limited to a specific taxon, genus, species, or organism [7]. When compared to non-natural compounds, natural products offer high chemical diversity and improved specificity and binding efficiency, making them good candidates for cosmetic applications [40,41].

To adapt to the hostile conditions of the marine environment, marine organisms have developed different mechanisms leading to the production of different bioactive compounds [2]. Although organisms living in marine environments use these compounds for reproduction, communication, and protection purposes, many present great potential to improve humans’ health and well-being [2,42]. Marine natural products can be organized according to their chemical structures (i.e., mycosporine-like amino acids, polysaccharides, carotenoids, polyphenolic compounds, fatty acids, peptides, terpenes, and alkaloids), as seen in Table 1, or by the phylum that produces them (i.e., Porifera, Chordata, Cnidaria, echinoderms, Algae, bacteria, fungi, Arthropoda, and phytoplankton) [43]. It is very important for the selection of compounds for pharmacological and cosmetic purposes to correctly identify the taxon of macro- and microorganisms to facilitate the reproduction, replication, and isolation of bioactive compounds [42].

**Table 1:** Marine-sourced bioactive compounds with applications in cosmetics.

**Compound Class**	Compound	Findings	Bioactivity	Phylum	Refs.
Mycosporin-like amino acids (MAAs)	Porphyra-334	A cream containing both Porphyra-334 and Shinorine (Helioguard 365^®^, Aqua (and) Lecithin (and) Alcohol (and) Sodium Lactate (and) Porphyra Umbilicalis (and) Phenoxyethanol, Mibelle AG Biochemistry, Germany) improved keratinocytes cell viability and diminished DNA damage in a dose-dependent manner upon UVA irradiation in vitro. Human studies revealed that Helioguard 365^®^ reduced lipid perioxidation by 35% in a single application and, after a 4-week application, skin firmness and smoothness improved by 10% and wrinkle depth was reduced by 20%. Helioguard 365^®^ also presented in vitro anti-inflammatory activity by promoting or inhibiting anti- and pro-inflammatory mediators.	Photoprotective, anti-aging, antioxidant, and anti-inflammatory.	Rhodophyta	[44,45]
	Shinorine				
	Palythine	Palythine at 10% presented an in vitro SPF of 17.9. In vitro, it was shown to inhibit solar radiation-induced cell death of keratinocytes and DNA damage. Solar radiation-induced oxidative stress and pro-inflammatory gene expression were also reduced in vitro. A cream containing palythine (Helinori^®^, Water (and) Porphyra Umbilicalis Extract, Gelyma, France) prevented the formation of sunburn cells by 94% after three days of application, and protected fibroblasts and keratinocytes from UVA-induced oxidative stress in vitro.			[46,47]
	Mycosporine-glycine	Suppressed the expression of UV-induced age-related genes and inflammation-related genes in vitro. Reduced radiation-induced oxidative stress in vitro. Presented, in vitro, wound healing in keratinocytes. Inhibited lipid peroxidase in a dose-dependent manner in vitro, with moderate activity at 30 µM.		Rhodophyta Chlrophyta Chordata Ascomycota Cyanobacteria	[45,47,48,49]
	Mycosporine-2-glycine	Reduced inflammation in keratinocytes upon irradiation in vitro. Presented antioxidant activity similar to ascorbic acid, in vitro and in vivo. Higher free radical scavenging activity than Trolox, determined by ABTS assay. Was shown in vitro to protect against oxidative stress-induced DNA damage.		Cyanobacteria	[45,50,51,52,53]
	13-O-β-galactosyl-Porphyra	Presented high protection for keratinocytes against UVA and UVB in vitro.	Photoprotective		[2,54]
	Collemin A	Provided protection for keratinocytes against UVA and UVB in vitro. Prevented complete formation of erythema, in humans, when applied 15 min prior to irradiation at a concentration of 6 µg/cm^2^ in vivo.		Ascomycota	[2,55]
Polyssacharides	Alginates	Alginates present applications as gelling agents in cosmetics. Hydrogel formation, promoting wound healing.	Gelling agent, wound healing	Ochrophyta	[56,57]
	Chitin	Skin humectant and rejuvenant with many commercialized applications.	Moisturizers	Arthopoda	[56,58,59,60]
	Chitosan			Arthopoda Zygomycota Chlorophyta	
	Fucoidan	Promotes fibroblast proliferation and collagen deposition in vitro. Protects the elastic fiber network of the skin in vitro. Was shown in vitro to regulate MPPs’ activity and secretion. Inhibited tyrosinase activity and presented anti-melanogenesis activity in an in vitro B16 model.	Anti-aging, whitening, and wound healing.	Chlorophyta Rhodophyta Phaeophyta	[44,61,62]
	Carrageenan	Shown to scavenge free radicals and reduce DPPH levels in vitro. Provides photoprotection by diminishing ROS levels in UVB-irradiated keratinocytes in vitro. Injections of carrageenan resulted in removal of dermal melanin in guinea pigs. Applications as viscosifier and binder in toothpastes. Facilitates and improves water absorption by the skin.	Antioxidant, anti-photoaging, and whitening.	Rhodophyta	[63,64]
	Hyaluronic acid	Forms a film in the stratum corneum that not only protects the skin but also prevents transepidermal water loss and moisturizes the epidermis. Vastly present in many commercialized formulations.	Skin rejuvenant, wound healing, and anti-aging.	Gyrista	[56]
	Gracilaria lemaneiformis polysaccharide fraction 2 (GLP-2)	Promotes keratinocytes proliferation during in vitro wound healing assays.	Wound healing	Rhodophyta	[62,65]
	Laminarin	Presents high DPPH scavenging activity and potent ROS absorbance capacity in vitro. Protects against UVB damage by regulating MMP activity in vivo.	Antioxidant, anti-aging, and anti-photoaging.	Ochrophyta	[61,66]
Carotenoids	β-carotene	Delays skin aging by reducing the expression of MMP and, consequently, extracellular matrix degradation, in vitro, upon irradiation or when not irradiated. Protects against UVA damage in vitro.	Anti-aging, antioxidant, and photoprotective.	Chlorophyta Tracheophyta Cyanobacteria Mollusca Arthropoda Echinodermata Euryarchaeota	[67]
	Astaxanthin	Very strong antioxidant activity. Reduced the production of melanin in vitro.	Antioxidant, anti-photoaging, and whitening.	Chlorophyta Pseudomonadota	[27,68,69]
	Fucoxanthin	Strong antioxidant activity and protective effect against oxidative stress in vitro using Vero cells. Significantly reduced UV-induced DNA damage in vitro. Presented in vivo antioxidant activity, translated in the decrease of lipid peroxidation.	Antioxidant and photoprotective.	Ochrophyta	[27,70,71]
	Zeaxanthin	Presented activity against UVB-induced skin damage in vivo. Inhibits tyrosinase in vitro.	Antioxidant, photoprotective, and whitening.		[71,72]
Polyphenols	Dieckol	Shown to promote hair growth in mice in vivo. Inhibits hyaluronidase in vitro.	Hair growth and anti-aging.		[73,74]
	Phlorotannins	Protected HaCat from UV-induced apoptosis in vitro. Inhibitors of tyrosinase and hyaluronidase in vitro.	Whitening, anti-aging, and photoprotective.		[74,75]
	Sargachromanol E	Inhibition of MMPs after UV irradiation of dermal fibroblasts in vitro. Anti-melanogenic activity in vitro.	Anti-photoaging and whitening.		[76]
Fatty Acids	Omega-3 polyunsaturated fatty acids	With dietary consumption of these fatty acids, the production of proinflammatory eicosanoids is decreased upon UV irradiation. Suppress UV-induced keratinocyte damage in vitro. Shown to reduce erythema and polymorphic light eruption in humans when taken orally. Accelerated wound healing in vivo when administered by IV in rats.	Anti-photoaging, anti-aging, and wound healing.	Chordata	[77]
	Docosahexaenoic acid	Increased photoprotection by increasing resistance to UV-induced apoptosis and reducing proinflammatory mediators in human keratinocytes in vitro. Prevents UV-induced photoaging by reducing MMPs in vitro. Reduced erythema and proinflammatory factors in mouse skin upon irradiation in vivo. Decreased erythema in human skin in vivo. Accelerated wound healing in mice by modulating inflammation in vivo.	Anti-photoaging, anti-aging, and wound healing.		[77,78,79]
	Eicosapentaenoic acid	Reduced inflammation mediators upon irradiation in keratinocytes and fibroblasts in vitro. Modulated MMPs’ expression in dermal fibroblasts upon irradiation in vitro. Oral administration suppressed ear edema upon irradiation in mice in vivo. Prevents skin moisture loss.	Anti-photoaging, anti-aging, photoprotective, and moisturizer.		[76,79,80,81]
	Linoleic Acid	When administered orally or topically, reduced UV-induced skin erythema in hairless mice in vivo. Improved wound healing when applied topically by reducing the thickness of the necrotic cell layer in vivo.	Anti-photoaging, photoprotective, anti-aging, and wound healing.		[77]
	α-Linoleic Acid				
	Omega-6 polyunsaturated fatty acids	Modulates and/or enhances inflammatory infection in the wound, accelerating wound healing. Restores transepidermal water loss.	Wound healing and moisturizer.	Ochrophyta	[68,77,82]
Proteins and Peptides	Collagen	Enhances skin regeneration in wounds when incorporated into dressings in vivo. Reduces skin pigmentation by inhibiting tyrosinase. Modulates MMPs. Prevents photoaging in mice irradiated with UV rays by modulating induced oxidative stress in vivo.	Whitening, wound healing, anti-aging, antioxidant, and anti-photoaging.	Mollusca Chordata Porifera Cnidaria	[83,84,85,86,87,88,89]
	Carnosine	Presented in vitro antioxidant activity.	Antioxidant	Chordata	[83,90]
	Anserine				
	Gelatin	Reduced photoaging in mice skin and suppressed UV-induced injury upon irradiation in vivo.	Anti-photoaging and photoprotective.		[83]
	Gelatin peptides				
	Aosa biopeptide	Stimulates collagen production in fibroblasts in vitro.	Anti-aging	Chlorophyta	[90]
	B-phycoerythrin	Used as pigments in makeup formulations and other cosmetics.	N/A	Rhodophyta	[91]
Alkaloids	Golmaenone	Presented radical scavenging activity and protected from UVA radiation in vitro.	Antioxidant and photoprotective.	Ascomycota	[16,92]
	Dihydroxyisoechinulin A				
	Echinulin				
Terpenoids	Tetraprenyltoluquinol	Reduced ROS production in fibroblasts in vitro.	Anti-photoaging, antioxidant, and photoprotective.	Ochrophyta	[93]
	Spatane diterpenoids	Induces apoptosis on cells that suffered photodamage in vitro.	Photoprotective		[31,93]

### 4.1. Marine Natural Products by Compound Class (Table 1)

#### 4.1.1. Mycosporine-like Amino Acids (MAAs)

Mycosporines are small, water-soluble molecules composed of either an aminocyclohexenone or an aminocycloheximine ring, carrying nitrogen or amino alcohol substituents. When swapped with amino acid residues, they are designated mycosporine-like amino acids (MAAs) [94]. The production of MAAs by marine organisms (i.e., cyanobacteria, phytoplankton, lichens, gorgonians, cnidarians, sponges, shrimp, sea urchins, starfish, clams, ascidians, and marine algae) is promoted by high UV stress. MAAs act as UV shields by absorbing and dissipating UV radiation in the form of innocuous heat without the risk of originating photochemical reactions [12,15]. Moreover, MAAs present high antioxidant activity [16]. Daniel et al. found that the UVA photodamage-neutralizing capacity of a cream containing 0.005% marine-derived MAAs was comparable to a cream containing 1% of a synthetic UVA and 4% of synthetic UVB filters [95]. Waditee-Sirisattha et al. applied MAA-containing emulsions (i.e., mycosporin-2-glycin, shinorine, or porphyra-334) to the ears of mice at a dose of 50 mg/ear to assess their ability to protect against UV-induced photooxidative stress. Results showed that although the MAA-containing emulsions did not protect against the accumulation of glycation end-products, the activities of antioxidant enzymes were upregulated, and thus the emulsions presented anti-photooxidative abilities upon irradiation of the mice’s ears [96]. Other studies suggested the photoprotective capacity of other marine-derived algae, such as red algae *Acanthophora spicifera (M.Vahl) Børgesen* and *Asparagopsis armata Harv*., and their potential to be included as ingredients for skin care products due to their photoprotective and anti-aging properties [65,97,98]. Currently, there are some commercialized products formulated with marine-derived MAAs aiming at photoprotection and anti-aging effects [13,16].

#### 4.1.2. Polysaccharides

Polysaccharides are desirable cosmetic ingredients due to their high water retention and anti-inflammatory and antimicrobial abilities, and the fact that they are produced by different marine organisms, such as those from the phyla Crustacea, Phaeophyta, and Rhodophyta [16]. Currently, there are two classes of marine-derived polysaccharides with industrial applications derived from Kappaphycus, Gigartina, Chondrus, and many other species: sulfated polysaccharides (e.g., fucoindans, carragenans, laminarans, galactans, and ulvan) and nonsulfated polysaccharides (e.g., alginates and agars) [31,99,100]. The most important marine source of polysaccharides is algae [101]. Xie et al. isolated 3,6-anhydrogalactose from different red algae and demonstrated that polysaccharides isolated from *Porphyra haitanensis*, *Gracilaria chouae*, and *Gracilaria blodgettii* present potential skin whitening and tyrosinase-inhibiting activities [98]. Moreover, red algae-derived polysaccharides also present other properties of cosmetic interest, such as wound healing. Veeraperumal et al. studied polysaccharides derived from *Gracilaria lemaneiformis* (GLP fractions). Results demonstrated that GLP fraction 2 (GLP-2) promoted wound healing through the enhancement of cell proliferation and migration of keratinocytes, demonstrating the potential for incorporating GLP fractions into wound healing products [65]. Polysaccharides isolated from brown seaweed also present properties with cosmetic applications. Jesumani et al. isolated polysaccharides from *Sargassum* sp. with skin whitening (tyrosinase-inhibiting ability), anti-aging (elastase inhibiting ability), antioxidant (antioxidant and scavenging abilities), and moisturizing and water retention properties, demonstrating *Sargassum* sp.-isolated polysaccharides’ potential to be included in cosmetic formulations [102]. Fucoidan is a sulfated polysaccharide also derived from brown algae. As previously discussed in the case of hyaluronic acid, fucoidan’s bioactivities vary according to its molecular weight. Regarding skin whitening abilities, low-molecular-weight fucoidan seems to be the most suitable, as the skin whitening properties of this polysaccharide seemed to increase as its molecular weight decreased, translated by increased tyrosinase-inhibiting, antioxidant, and cellular melanogenesis-inhibiting properties [103]. Algal polysaccharides are also known for their enhanced moisturizing properties (i.e., chitin, chitosan, and hyaluronic acid) [104,105]. Data obtained from clinical studies validated that polysaccharide-based cosmetic formulations reduced transepidermal water loss and improved the skin barrier’s structural integrity [106].

#### 4.1.3. Carotenoids

Carotenoids are naturally occurring pigments with photosynthetic light-harvesting properties, transferring absorbed solar energy to chlorophylls and acting as photoprotectors and photosynthetics [101]. These compounds can be found in the cells of cyanobacteria, but are also present in other marine organisms, and protect these cells against the photooxidative stress generated by photosynthetic metabolism [107,108]. Pagels et al. studied the cosmetic potential of *Cyanobium* sp. carotenoids that could be incorporated into anti-aging formulations due to their anti-hyaluronidase properties [107]. Carotenoids-rich *Padina australis* extracts, algae found in Malaysian waters, were prepared in the work of Thiyagarasaiyar et al., and their antioxidant and photo-shielding abilities were assessed. Results showed that these carotenoid-containing extracts were a promising source of antioxidants, demonstrated by the DPPH and reducing power assays, and, upon UV radiation, they protected human keratinocytes from UV-induced damage [109]. Jeong et al. also explored the antioxidant properties of the yellow carotenoids Xantophylls and confirmed their bioactivity in DPPH and ABTS assays [110].

Although many marine-derived carotenoids present high antioxidant properties, as explored, few are present in commercialized cosmetics and sunscreens as clinical trial results were disheartening. Skin absorption of marine carotenoids is yet to be determined and is crucial to proving their potential as antioxidant ingredients in sunscreen formulations [13,111].

#### 4.1.4. Polyphenolic Compounds

Polyphenols (i.e., phenolic acid, flavonoids, tannins, and phlorotannins) are secondary metabolites that are involved in many biological processes, such as reproduction, photosynthesis, and cell division, and present antioxidant and anti-melanogenesis properties [31,97]. Polyphenolic compounds sourced from marine algae (i.e., dieckol, phloroglucinol, fucofuroeckol-A, and triphlorethol-A) also present photoprotective abilities, especially against UVB rays, thus presenting potential to be included in sunscreens [101]. In particular, phlorotannins, the most studied class of polyphenolic compounds found in seaweed, also present a vast range of bioactivities, such as skin whitening, antioxidant activities, UV protection, anti-aging, and are consequently being researched for cosmetic applications [112]. Jesumani et al. extracted a polyphenol-rich fraction from *Sargassum vachellianum* and compared its antioxidant, photo-shielding, anti-photoaging, and anti-aging properties to a polysaccharide-rich fraction extracted from the same algae. Results showed that the polyphenol-rich fraction presented higher free radical scavenging ability and effectively absorbed UV (UVA and UVB) radiation compared to the polysaccharide-rich fraction [113]. Arguelles et al. evaluated the total polyphenolic content of *Turbinaria ornata* and confirmed the antioxidant activity and very high tyrosinase-inhibiting activity of these phenolic compounds, demonstrating their potential for anti-aging and skin whitening formulations [114]. Castejón et al. also demonstrated the potential of polyphenol fractions for inclusion in anti-aging formulations [115]. They produced extracts from Icelandic seaweeds and demonstrated the enzymatic-inhibiting activities of collagenase, elastase, hyaluronidase, and tyrosinase, thus presenting anti-aging and skin whitening properties Moreover, Vega et al. demonstrated the UV-absorbing abilities of polyphenols and, consequently, their potential to be included in sunscreen formulations for protection against broad-range UVs [116]. Soleimani and colleagues also demonstrated the potential of polyphenolic compounds for sunscreen formulations. In their work, the ethyl acetate fraction of *Padina boergesenii* was isolated and its antioxidant and UV-shieling effect upon UV-induced damage of keratinocytes was demonstrated, highlighting the potential of polyphenolic compounds as UV shields [117].

#### 4.1.5. Fatty Acids

Fatty acids are known dietary supplements that also present a broad spectrum of properties for topical applications in cosmetics, such as soft tissue repair and skin nourishment by stimulating collagen production, as well as anti-inflammatory and wound healing properties. Among the different fatty acids, polyunsaturated fatty acids (PUFA), specifically the omega-3 fatty acids docosahexaenoic acid (DHA) and eicosapentaenoic acid (EPA), have been linked to several health benefits, such as healthy aging [16,118]. The main source of omega-3 fatty acids for humans is through the dietary consumption of fish; however, fish shortages and increased costs have encouraged the search for alternative sources of DHA and EPA (i.e., plants, algae, bacteria and fungi). When compared to fish oil, fatty acids derived from single-cell organisms, such as yeast and mold, and denoted single-cell oils (SCOs), present higher oil content and an increased number of antioxidant molecules, such as carotenoids [16,119,120]. Fish oil-derived omega-3 fatty acids are known to improve skin health when orally ingested [13,121]. For example, the squid *Loligo loligo* is considered a new source for omega-3 and omega-6 oils, with a high percentage of linoleic acid, EPA, and DHA [13,122]. Although the aquaculture of these species is yet to be explored, aquaculture of *Octupus vulgaris* as a source of fatty acids has proven preliminarily successful, supporting further research for other promising species [13].

#### 4.1.6. Proteins and Peptides

Collagen, the main structural protein of connective tissue, can be found in many cosmetic products due to its humectant, moisturizing, anti-aging, anti-wrinkling, and skin repairing properties [123,124]. However, proteins with high molecular weight, as in the case of collagen, cannot cross the skin barrier, remaining at its surface (the *stratum corneum*) [84,123]. Usually, the collagen present in commercialized cosmetic formulations is from bovine origin, resulting in regulatory and ethical issues. Marine collagens can be seen as an alternative to bovine collagen as they can be derived from fish and Porifera, Echinodermata, Cnidaria, and Mollusca phyla organisms, with some presenting increased biocompatibility and mechanical strength. However, these collagens also present lower degradation temperature, limiting their application [13,27,84,124].

#### 4.1.7. Alkaloids

Alkaloids (i.e., pyridoacridine, indole, pyrrolizidine, isoquinoline, guanidine, amino imidazole, and steroidal alkaloids) can be derived from many marine organisms (e.g., sponges, tunicates, anemones, algae, and mollusks) and present antioxidant and anti-inflammatory activities [7,43,125]. Potential applications of marine-derived alkaloids in cosmetic formulations include sunscreens and facial moisturizers and creams [126]. Studies highlighting the cosmetic potential of alkaloids have shown that daily application of an alkaloid-containing facial cream improved the skin’s appearance in patients with mild to moderate rosacea by 70%. Moreover, 50% of the participants involved in a study self-reported, in Patients Global Assessment, improvements in skin appearance after 30 days of application [127]. Hwang et al. identified the photoprotective effect of topsentin, an alkaloid isolated from the marine sponge *Spongosorites genitrix*, and demonstrated that topsentin protected human keratinocytes against UV-induced damage, signifying its potential for cosmetic formulations [128]. Viridicatin and viridicatol are two alkaloids isolated from *Penicillium echinulatum* by Teixeira and colleagues, who demonstrated their UV-absorbing and anti-photooxidative stress properties in human keratinocytes [129].

#### 4.1.8. Terpenoids

Terpenes and terpenoids are aromatic hydrocarbon molecules found in most plants and can be used as replacements for artificial scents and flavors. This class of compounds presents many biological activities, such as antioxidant and anti-acne properties [81,130,131]. The bactericidal activity against *P. acnes* of marine-derived terpenes, such as sargafuran, has been compared to clindamycin, used to treat acne, as part of efforts to minimize the changing resistance mechanisms of bacteria [32]. Balboa et al. isolated a tetraprenyltoluquinol meroterpenoid from *Sargassum muticum* and demonstrated that this terpenoid was able to attenuate UVA-induced damage in vitro and protected against ROS production, with antioxidant activity comparable to retinoic acid. This demonstrates the potential of terpenoids to be included in cosmetic formulations for their antioxidant and anti-photoaging properties [132].

### 4.2. Marine Natural Products by Phylum

#### 4.2.1. Porifera

Marine sponges are invertebrates belonging to the phylum Porifera that can be found attached to the ocean floor. They produce several compounds in order to block the growth of and attacks from intruding species, repel predators, and attract food [133]. Thus, marine sponges are a very rich reservoir of natural products with diverse chemical structures and bioactive properties, including antimicrobial properties. For sponge symbiosis, survival, and metabolite production, microbes produce novel chemicals useful for cosmetics [134,135]. Through gene and enzyme engineering, pathways reconstructing, and metabolic networks, these microbes can be modified to exploit the production of compounds of interest [134].

#### 4.2.2. Chordata

Ascidians, or sea-squirts, from the phylum Chordata, encompass more than 3000 species that usually form symbiotic relations while living in protected areas [2]. As a chemical defense mechanism against predators and infectious microbes, many ascidians can accumulate heavy metals, and are very useful ecologically to protect adverse effects in aquaculture. The most common natural products produced by ascidians include alkaloids and peptides [136,137].

#### 4.2.3. Cnidaria

The Cnidaria phylum has been overlooked in terms of natural products development as it has been less explored than other phylums. The main source of known natural products in this phylum are benthic cnidarians, but some have also been extracted from pelagic cnidarians, including scyphomedusae. These species of Cnidaria present collagen, fatty acids, and other compounds extracted from their crude venom, such as glyco- and phosphoproteins [2,138]. Although scyphomedusae significantly interfere with human activity along the coast, they present many health benefits when included in diet, and are very common in China and other countries in Southeast Asia (e.g., *Rhopilema esculentum*, *Rhizostoma pulmo,* and *Aaurelia aurita*). Many studies are broadening knowledge on the health benefits of Cnidaria as food supplements, cosmetic ingredients, and for biomedical and biomaterial applications, mainly due to their antioxidant and anti-photoaging activities [42,101,139].

#### 4.2.4. Echinoderms

The Echinodermata phylum includes many known sea animals, such as different species of starfish and sea cucumbers. Sea cucumbers present unique wound healing properties and are commercially used in the form of dried powders and extracts [1,132,140]. On the market, sea cucumbers and their compounds can be commonly found in food supplements, toothpaste, ointments, body lotions, and facial skin cleansers [140]. For example, *Stichopus hermanni* presents wound healing properties by accelerating the rate of wound contraction, which is attributed to its high content of proteins, glucosaminoglycans, chondroitin sulphate, growth factors, and fatty acids, such as EPA and DHA [141]. *Stichopus choronotus* is another species of sea cucumber known to promote wound healing [2]. Extracts of sea cucumbers have been incorporated in Carbopol^®^ gel and topically applied for 12 weeks to diabetic foot ulcers in order to assess their skin soothing potential. Sea cucumber extracts have also been associated with anti-inflammatory properties due to their high saponin content, inhibiting the production of tumour necrosis factor (TNF-α) [142].

#### 4.2.5. Algae

The algae taxon, known as macroalgae or seaweeds, are aquatic photosynthetic organisms that include Rhodophyceae (red algae), Phaeophyceae (brown algae), and Chlorophyceae (green algae) phyla [27,143]. Seaweeds are the most extensively studied marine organisms since they are a biodegradable and non-toxic source of natural compounds with a vast array of bioactivities, such as delaying skin aging, antioxidant activities, and immunomodulatory activities [2,92]. Seaweeds are also known for their high polysaccharide content (e.g., fucoidan), with, for example, antioxidant and anti-inflammatory assets, which can be applied to many products, including hydrocolloids. Algae also present high levels of other important bioactive metabolites, such as polyphenols, phlorotannins, carotenoids, and vitamins [2,27,92]. The red algae species *Corallina officinalis* has cosmetic applications due to acting as a shield against UV and infrared radiation and antioxidant activity [144]. Brown macroalgae, of the Phaeophyceae phylum, are used in cosmetics as a source of vitamins, amino acids, minerals, lipids, sugars, and other compounds. For example, the brown algae *Macrocystis pyrifera* is used in cosmetics as a thickening agent and emulsion stabilizer [145]. Another brown algae that is less explored but holds interesting properties for cosmetic applications is *Ecklonia maxima*, with antioxidant, anti-melanogenesis, and photo-shielding properties [11]. Green algae are also used for cosmetic applications as pigments or as a source of phenolic compounds, sterols, and vitamins. Thus, ingredients sourced from Chlorophyceae can be found in moisturizers, anti-stretch mark creams, eye balms, face masks, anti-aging products, sunscreens, scrubs, face peelers, firming ointments, purgative gels, etc. [146].

Microalgae are microscopic, photosynthetic, unicellular organisms that produce a wide range of proteins, lipids, carbohydrates, carotenoids, and vitamins with biological activity. The microalgae genera *Arthrospira* and *Chlorella* are vastly explored in the cosmetic industry, with their extracts being used as ingredients for face and skin care products. Microalgae mainly present antioxidant, anti-aging, skin refreshing, and regeneration properties, but can also act as photo-shielding agents and can be incorporated into hair care products [3,144]. There are currently several commercialized cosmetic products using microalgae, such as Dermochlorella DG^®^ from CODIF Research & Nature, a *Chlorella* sp. extract with oligopeptides, XCELL-30^®^ from Greensea using microalgae endemic to Madagascar, and Alguronic Acid^®^ from Algenist and Alguard^®^, a polysaccharide compound isolated from *Porphyridium* sp. [2,3,13,27]. Commercialized species of microalgae include *Spirulina*, *Chlorella*, *Haematococcus*, *Dunaliella*, *Botryococcus*, *Phaeodactylum*, and *Porphyridium* [147]. Cosmetic products that use pigments sourced from algae include eye shadow, face make-up, and lipsticks [143,144].

#### 4.2.6. Bacteria

As previously discussed, microorganisms, such as bacteria, can be a sustainable and economic alternative for sourcing of MAAs, carotenoids, and fatty acids used in cosmetics. For instance, *Pseudomonas* sp. has been shown to produce methylene chloride, a known tyrosinase inhibitor and skin whitening agent [16].

#### 4.2.7. Fungi

Recently, diverse marine fungi have been discovered in regions ranging from the coast to the deep sea. Naturally, due to being less accessible, deep-sea fungi have been less explored, but seem to be relevant and abundant sources for bioactive molecules for cosmetic development [16]. For instance, the genus *Acremonium*, found in sponges, mangroves, and seawater, was shown to produce derivates of hydroquinone with significant antioxidant activity that was found to be higher than that displayed by synthetic hydroquinone derivates, such as butylated hydroxytoluene (BHT) [16].

#### 4.2.8. Corals

Corals are not a novelty for cosmetics. They have been extensively used for years in the form of powders (for example, as skin scrubbers) and as mineral suppliers due to their mineral content and overall good textural and physicochemical characteristics. Moreover, coral powder has also been used due to its photo-shielding, antioxidant, anti-aging, and anti-acne properties [147].

#### 4.2.9. Phytoplankton

Phytoplankton are a very diverse group of important organisms that include, among others, diatoms, cyanobacteria, and dinoflagellates. They are very rich in lipids and omega-3 fatty acids [147,148]. The production of pro-ceramides by cells is stimulated and the protective barrier of the skin is renewed. Thus, phytoplankton can be used for their skin whitening and toning, anti-wrinkling, and anti-aging properties [26,147]. For instance, extracts of cyanobacteria have attracted interest for research for the development of cosmetics targeting the prevention of skin aging [149].

#### 4.2.10. Sea Water and Sea Mud

Sea water is an abundant source of many minerals (e.g., potassium, magnesium, sulphates, sodium, calcium, and chlorides), with known benefits that are used for skin care cosmetics [150]. In particular, deep-sea water, which is found in extreme conditions (i.e., absence of or very little light, near-freezing temperatures, and high pressure), presents properties with very high impact on skin health, especially in diseases, such as atopic dermatitis [150,151,152]. Such beneficial properties are thought to be related to an even higher mineral content than average sea water [153]. Examples of commercialized cosmetics incorporating sea water as ingredients include Pheohydrane^®^ [99].

Sea mud, also rich in many salts and minerals, presents antibacterial activity against many bacterial strains (e.g., *Escherichia coli*, *S. aureus*, *P. acnes*, and *Candida albicans*) [152,153] and has been incorporated into many cosmetic formulations, proving especially useful for conditions, such as psoriasis [37,99,154,155]. It has been shown to balance skin pH, promote skin repair and hydration through water retention, and has preventive properties towards acne and aging. Sea mud has also been incorporated in face masks, where it has been shown to promote skin tightness while cleaning and invigorating the skin [37,99,155].

Unfortunately, both sea water and sea mud may contain naturally occurring toxic metals, such as cadmium and lead. As such, to be incorporated in cosmeceuticals or other products, strict control must be enforced [37,99,154,156]. Thus, products containing sea water or mud must include clear product labels informing consumers of the risks associated with such elements, especially for consumers with sensitive skin. Cosmetic products containing both sea water and mud in their composition have already reached the market, such as the Ahava^®^ range of products and Erno Laszlo^®^ [15,37,99].

## 5. Challenges and Opportunities

**Figure 2:**
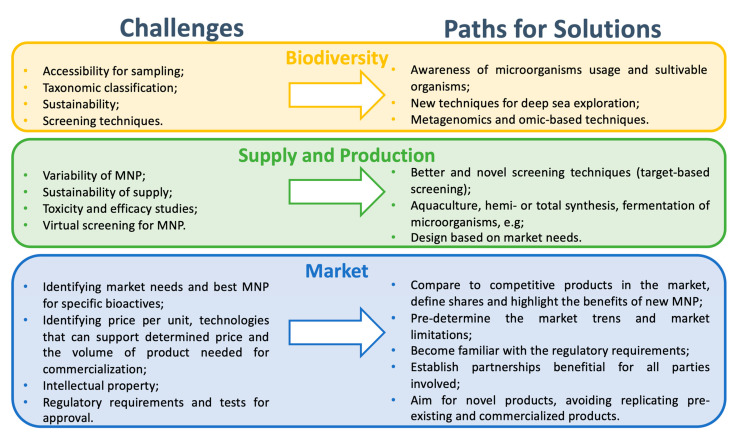
Challenges faced by marine natural products (MNP) to enter the market.

The questions to be addressed and the challenges faced by the commercialization of marine-derived cosmetic products are briefly summarized in Figure 2. The main challenges faced by cosmetic formulations relate to their overall sustainability, and include production capacity, ecotoxicity, economic value, and generated waste. Challenges in using marine-derived compounds as ingredients for cosmetic formulations arise from the level of standardization, efficacy, and traceability, and can be categorized into three main categories: accessibility and efficient screening; sustainable production of the bioactives and knowledge of their mechanism of action; and market issues, such as processes, costs, and partnerships.

In the case of marine-derived ingredients, it is imperative to evaluate the absence of heavy metals that occur naturally in marine ecosystem (e.g., cadmium, lead, arsenic, and mercury), as well as toxins and allergens [157]. Moreover, marine-derived compounds also face the challenge of volume of production, as compounds are usually present in small quantities and are often challenging to isolate [7]. It is also challenging to ensure that marine-derived compounds do not change, especially marine-derived metabolites, as their environmental conditions are not static [158]. A useful strategy to overcome these challenges is hemi-synthesis, in which instead of harvesting the compound of interest, its natural source is harvested and converted into the compound of interest [7]. Sourcing marine-derived compounds also faces the challenge of limited accessibility, especially if the target organisms inhabit deep ocean floors. However, over the years, not only scuba diving techniques but also submersible equipment, such as remotely operated vehicles, have allowed for observation and sampling of marine organisms that live in otherwise inaccessible environments [7,16,42,133].

Another concern related to the high demand for personal care products and cosmetics worldwide is the ecotoxicity displayed by synthetic compounds in such products [159]. Natural ingredients have been described as a substitute for synthetic ingredients because when returned to the environment these compounds are generally less hazardous [160]. Ecotoxicity has been a concern tightly related to sunscreens containing synthetic UV filters [161], and throughout this review, the high efficacy of marine-derived compounds as photo-shielding agents has been discussed. Moreover, the high volume of commercialization of cosmetic products also raises ecotoxicity concerns regarding the packaging used and the resulting waste, as waste resulting from overall packaging has significantly contributed to land and marine pollution [9]. Regarding the marine environment, plastic has been found to be the main pollutant, representing 50–90% of marine debris. In 2018 alone, 7.9 billion units of plastic debris attributed to cosmetics and personal care products entered US seas [162,163]. Sustainably produced naturally sourced materials have therefore been introduced as materials for packaging cosmetic products [164]. An example of such packaging is compostable packaging [165]. Marine-sourced materials that can be used for zero-waste packaging of cosmetics are edible materials (e.g., seaweed), creating edible packaging. Seaweed is a prime example of marine-derived packaging materials as it presents high fiber and vitamin content and can be made into packaging without the need for chemicals. However, applications for seaweed-based packaging are currently limited to food products, such as sandwich and burger wrappers and instant coffee and seasoning plastic sachets to replace single-use plastics [166]. The questions to be addressed and the challenges faced by the commercialization of marine-derived cosmetic products are briefly summarized in Figure 2.

## 6. Regulatory Requirements

Marine-derived compounds can be used for many applications (i.e., food supplements, medicines, and cosmetics). In the case of food supplements and medicines, products are strictly regulated in part through their application. When the applicant is deciding the category into which a product will fall, safety, efficacy, and quality requirements must be known and very well characterized [10].

Because it is highly dynamic, complex, and fast-paced, the cosmetic sector must also be regulated. Regulatory frameworks around the world are relatively similar, even if several differences still exist [10,167,168,169,170]. In the US, there are two important laws related to cosmetic products regulated by the Food and Drugs Administration (FDA)—the Federal Food, Drug and Cosmetic Act (FD&C Act) and the Fair Packaging and Labeling Act (FPLA). In Canada, the Cosmetic Regulation Act (1977) and Food and Drugs Act (1985) have undergone a few amendments over the years. In Brazil, the cosmetic sector is regulated by three authorities: the Ministry of Health, the Brazilian Health Regulatory Agency (ANVISA), and Hygiene, Perfume, Cosmetics and Sanitizing Products Management (GHCOS). In Japan, cosmetic products are regulated under the Pharmaceutical and Medical Devices Law (PMDL) by the relevant authority, the Ministry of Health, Labor and Welfare. In China, there are three major authorities: the State Administration for Market Regulation (SAMR), the National Medical Products Administration (NMPA), and the General Administration of Customs (GAC). The new cosmetic regulation (Cosmetic Supervision and Administration Regulation (CSAR) implemented in 2021) replaced the Cosmetics Hygiene Supervision Regulations (CHSR). Several subsidiary regulations have been announced, notably related to the registrations and notification process, GMPs, and monitoring of adverse reactions. Regarding the European Market, Regulation (EC) N° 1223/2009 is the main regulatory framework for finished cosmetic products [168].

According to Regulation (EC) N° 1223/2009 [168], “a cosmetic product made available on the market shall be safe for human health when used under normal or reasonably foreseeable conditions of use”. This regulation strengthens the safety of these products and streamlines the framework for all operators in this sector. In fact, safety is an important issue for any cosmetic, independent of the origin of the ingredients, including marine-derived compounds. A Cosmetic Product Safety Report (CPSR) is mandatory for every cosmetic product and included in the Product Information File (PIF). The CPSR consists of two parts that should contain the following information: cosmetic product safety information (Part A) and cosmetic product safety assessment (Part B). Both the CPSR and PIF must be kept updated.

According to Regulation (EC) N° 1223/2009 [168], “safety is based upon safe ingredients (toxicological profile, chemical, structure, exposure” and “the responsible person shall, prior to placing a cosmetic product on the market, ensure that the cosmetic product undergone a safety assessment on the basis of the relevant information and a cosmetic product safety report is set up”.

Animal testing in cosmetics has been widely discussed over the years, and it has been banned in several countries. Thus, raw material safety must be evaluated by using validated alternative methods. In vitro testing methods should preferentially be used and can include irritation and corrosivity for skin and eyes (EpiDerm (OECD 439) and EpiOcular (OECD 492) for skin and eyes, respectively), mutagenicity/genotoxicity (Ames Test (OECD 471) for mutagenicity and chromosome aberration test (OECD 473) for genotoxicity) and photo-induced toxicity (3T3 NRU). For in vivo safety testing, a human repeated insult patch test (HRIPT) can be used.

Hazard and exposure assessment of any chemical (in the macro-, micro-, or nano-scale) brought to the European market is subject to the REACH (Registration, Evaluation and Authorization of Chemicals) regulation (Regulation (EC) N.º 1907/2006) [164].

Regulation N° 1223/2009 also stipulates that all cosmetics must be manufactured in accordance with the harmonized standards laid out in Good Manufacturing Practices (GMPs). These GMPs ensure that all products are prepared in a clean environment and are not contaminated in production.

Moreover, the declaration of ingredients on finished cosmetic products must be in accordance with the International Nomenclature of Cosmetic Ingredients, the INCI System, and before being placed on the European market, all cosmetics products must be listed in a centralized database, the Cosmetic Products Notification Portal (CPNP), managed by the European Commission. This ensures the online availability of cosmetic information to authorities (that is, poison centers (or similar EU bodies)) for purposes of market surveillance and to prepare a plan of action in case of unexpected complications.

This EU Cosmetics Regulation also includes a set of strict rules for labelling, which must be present on the product container, packaging, or, if not possible given space restrictions, in an enclosed leaflet. This EU Cosmetics Regulation also sets the principles for claims that manufacturers can make on their packaging.

In general, a cosmetic ingredient dossier (CID) includes the raw data for all ingredients (evaluating their safety), a technical data sheet (TDS), a material safety data sheet (MSDS), and allergen declarations. It can also include the certificates of analysis (CoA), certificates of conformity, free-from certificates, and efficacy studies.

## 7. Conclusions

Consumer demand is turning to natural products, driving researchers to uncover alternative sources of bioactives and ingredients for cosmetics. The variety of molecules and compounds described in this review highlight the marine environment as an underexploited resource, especially for deep-sea-inhabiting marine organisms that remain to be described. Moreover, this review also points to the potential of marine-derived compounds to be included in cosmetics not only as the bioactive ingredient, but also as an ingredient (i.e., to increase viscosity or modulate the texture of the product). Once valuable species are clearly identified, it will remain to optimize the mode of extraction of the molecules of interest and to ensure their effectiveness and safety for cosmetic applications, which are endless. Bioactive compounds can increase protection against UV exposure, improve skin condition, and prevent skin aging. These ingredients should also be considered innovative compounds, as there are no terrestrial equivalents, and they will without a doubt bring benefits. However, the commercialization of cosmetic products containing marine-derived bioactive ingredients faces important challenges that must be carefully addressed. Furthermore, the safety and toxicology of cosmetic products containing marine-derived bioactives must be thoroughly analyzed and ensured, as “naturally occurring” does not translate to “safe for use”. As a future perspective, fruitful cooperation among researchers and cosmetic industries will drive this sector towards being more ecological through the responsible sourcing of ingredients, implementing earth-friendly manufacturing processes, and experimenting with inventive recycling and reuse programs, therefore contributing to improving our environment.

## Data Availability

No new data were created or analyzed in this study. Data sharing is not applicable to this article.
